# Childhood Trauma, Borderline Personality Disorder, and Inflammation: Specific Links of Emotional Neglect with CRP and IL-6 Elevation in BPD

**DOI:** 10.1192/j.eurpsy.2026.11007

**Published:** 2026-07-31

**Authors:** B. Dawidowski, L. Franczak, P. Podwalski, J. Samochowiec

**Affiliations:** Department of Psychiatry, Pomeranian Medical University in Szczecin, Szczecin, Poland

## Abstract

**Introduction:**

Childhood trauma has been linked to low-grade inflammation, but the contribution of trauma subtypes and the role of borderline personality disorder (BPD) remain unclear.

**Objectives:**

We assessed whether distinct trauma domains relate to inflammatory markers and whether differences between BPD patients and healthy controls (HC) shape these associations.

**Methods:**

In this cross-sectional cohort (N=90; BPD and HC), serum levels of CRP, IL-6, IL-1β, IFN-γ and TNF-β were measured. Trauma was assessed using TEC domains and summary scores. Linear models were fitted for each marker–trauma pair after Box–Cox/log transformations selected by AIC. Model diagnostics guided inference: if assumptions were met, classical t-tests were used; otherwise robust HC3 SEs and studentized wild bootstrap (B=2000). Multiple testing was controlled with Benjamini–Hochberg FDR. Additional sensitivity analyses added covariates (Group, BMI, education, smoking).

**Results:**

Between-group comparisons showed no differences for IL-1β, IFN-γ or TNF-β; these were not pursued further. After FDR correction, CRP was positively associated with emotional neglect (β≈0.09 on log-CRP; p=0.002; p_adj=0.018), robust to smoking adjustment. IL-6 correlated with total trauma load (p=0.005; p_adj=0.046) in simple models; however, this association attenuated after including diagnostic group, with BPD participants displaying higher IL-6 levels than HC regardless of trauma scores.

**Conclusions:**

Emotional neglect in childhood is specifically linked to elevated CRP, suggesting a pathway from relational deprivation to chronic low-grade inflammation. Elevated IL-6 appears more characteristic of BPD patients than of trauma exposure alone. Other cytokines (IL-1β, IFN-γ, TNF-β) showed no group differences. Findings highlight the clinical relevance of assessing emotional neglect, reducing modifiable risks such as smoking, and addressing inflammation in BPD.

**Disclosure of Interest:**

None Declared

